# Targeting the lysosome by an aminomethylated Riccardin D triggers DNA damage through cathepsin B‐mediated degradation of BRCA1

**DOI:** 10.1111/jcmm.14077

**Published:** 2018-12-18

**Authors:** Yanyan Wang, Huanmin Niu, Zhongyi Hu, Mengyuan Zhu, Lining Wang, Lili Han, Lilin Qian, Keli Tian, Huiqing Yuan, Hongxiang Lou

**Affiliations:** ^1^ Key Lab of Chemical Biology of Ministry of Education, Department of Natural Product Chemistry, School of Pharmaceutical sciences Shandong University Jinan China; ^2^ Institute of Medical Science The Second Hospital of Shandong University Jinan China; ^3^ Department of Biochemistry and Molecular Biology, School of Medicine Shandong University Jinan China; ^4^ School of Medicine Shandong Yingcai University Jinan China

**Keywords:** BRCA1, CTSB, DNA damage, RD‐N

## Abstract

RD‐N, an aminomethylated derivative of riccardin D, is a lysosomotropic agent that can trigger lysosomal membrane permeabilization followed by cathepsin B (CTSB)‐dependent apoptosis in prostate cancer (PCa) cells, but the underlying mechanisms remain unknown. Here we show that RD‐N treatment drives CTSB translocation from the lysosomes to the nucleus where it promotes DNA damage by suppression of the breast cancer 1 protein (BRCA1). Inhibition of CTSB activity with its specific inhibitors, or by CTSB‐targeting siRNA or CTSB with enzyme‐negative domain attenuated activation of BRCA1 and DNA damage induced by RD‐N. Conversely, CTSB overexpression resulted in inhibition of BRCA1 and sensitized PCa cells to RD‐N‐induced cell death. Furthermore, RD‐N‐induced cell death was exacerbated in BRCA1‐deficient cancer cells. We also demonstrated that CTSB/BRCA1‐dependent DNA damage was critical for RD‐N, but not for etoposide, reinforcing the importance of CTSB/BRCA1 in RD‐N‐mediated cell death. In addition, RD‐N synergistically increased cell sensitivity to cisplatin, and this effect was more evidenced in BRCA1‐deficient cancer cells. This study reveals a novel molecular mechanism that RD‐N promotes CTSB‐dependent DNA damage by the suppression of BRCA1 in PCa cells, leading to the identification of a potential compound that target lysosomes for cancer treatment.

## 1 INTRODUCTION

RD‐N, an aminomethylated derivative of riccardin D, is reported to be a potential anti‐cancer agent to induce cell death through lysosomal rupture. It was suggested that cathepsin B (CTSB) released from the lysosomes after RD‐N treatment triggered apoptosis in prostate cancer (PCa) cells,^1^ but the underlying molecular mechanisms responsible for the effects remained unknown.

Targeting lysosomes has great therapeutic potential in cancer, because it not only triggers apoptotic cell death pathways but also inhibits cytoprotective autophagy.^2^, ^3^ Lysosomal membrane permeabilization results in the release of lysosomal enzymes into the cytosol, which can initiate caspase‐dependent or ‐independent cell death. Cathepsin B is one of the key lysosomal cysteine proteases that play important roles in migration and invasion of human cancer cells.^4^, ^5^ On the other hand, cysteine cathepsins have been shown to mediate cancer cell apoptosis.^6^, ^7^ In addition to be localized in lysosomes, cysteine cathepsins and their splice variants are also detected and function in the nucleus,^8^, ^9^ plasma membrane^10^, ^11^ and extracellular milieu.^12^ In addition to being as primary lysosomal protein recycling machine, cysteine cathepsins are involved in multiple physiological and pathological processes through regulation of protein stability, initiation of proteolytic cascade and fusion with plasma membrane. For example, CTSB, once released from the lysosomes into the cytosol, induces cleavage of pro‐apoptotic factor Bid, which leads to cytochrome c release from mitochondria and ultimately caspase‐dependent apoptosis.^6^ However, CTSB‐mediated cell death is also observed in a caspase‐independent manner.^13^, ^14^ Despite considerable amounts of work published, the mechanisms underlying the activity of CTSB in the nucleus to induce cell death are largely unknown.

We have previously identified RD‐N, an aminomethylated derivative of bisbibenzyls Riccardin D, as a potential antitumour agent that was able to cause lysosomal membrane permeabilization.^1^ This study went a further step and showed that RD‐N‐induced cell death is significantly dependent on the translocation of CTSB to the nucleus after treatment. Pro‐apoptotic effect of CTSB, particularly the active enzyme domain of CTSB, is associated with suppression of breast cancer 1 protein (BRCA1) activity in the nucleus. Impairment of BRCA1 by CTSB facilitated DNA damage and cell death in response to RD‐N.

## 2 MATERIALS AND METHODS

### 2.1 Reagents

RD‐N was the aminomethylated derivative of riccardin D and its structure was identified as reported previously.^1^ The compound was dissolved in dimethyl sulfoxide (DMSO, Sigma‐Aldrich, St. Louis, MO) at 10 mmol/L as stock solution. E64d and z‐VAD‐fmk were obtained from Enzo Life Sciences. CA074Me was acquired from Calbiochem. Z‐RR‐AMC was purchased from the EMD Chemicals. Propidium iodide (PI) was purchased from Sigma‐Aldrich.

### 2.2 Cell culture and treatments

Human PCa cell lines PC3, DU145 and LNCaP were obtained from the American Type Culture Collection (Manassas, VA). All the cell lines were cultured in RPMI‐1640 (Hyclone, Logan, UT, USA) medium containing 10% foetal bovine serum (Gibco, Gaithersburg, MD, USA), 100 U/mL of penicillin and 100 μg/mL of streptomycin and maintained in a humidified incubator of 5% CO2 at 37°C. When growing cells reached approximately 50%‐70% confluence, they were treated with RD‐N or other chemicals as indicated. Vehicle treatment served as a control. Transient transfections were performed using Lipofectamine 2000 (Invitrogen). Prior to transfection, Lipofectamine (~2 μL/μg of DNA) was added to Optimum medium without foetal bovine serum. After a 5‐minute incubation, the mixture was then added on the top of the DNA constructs. After a 20‐minute incubation at 37°C, the whole solution was added on the cells. GFP‐tagged CTSB was a kind gift from Ted Hupp (University of Edinburgh).

### 2.3 xCELLigence

Experiments were carried out using the RTCADP instrument (Roche, Germany) which was placed in a humidified incubator maintained at 37°C with 5% CO2. For time‐dependent cell response profiling, 10 000 cells/well were added to 16 well E‐Plates. After 12‐16 hours PC3 cells were treated with the indicated compounds. The electronic sensors provided a continuous and quantitative measurement of cell index in each well. Cell index is a quantitative measure of cell number present in a well, for example, lower cell index reflects fewer cells are attached to the electrodes. The E‐Plate 16 was monitored over the time frame indicated.

### 2.4 Flow cytometry

We used Annexin V (0.1 mg/mL) for the assessment of PS exposure, and PI (0.5 mg/mL) for cell viability. Cell death was recorded in a FACScan cytometry (FACSCalibur, Becton Dickinson) in total population (10 000 cells/nuclei). For γH2AX detection, PC3 cells, untreated or RD‐N‐treated, were fixed in 4% paraformaldehyde, permeabilized with 0.1% Triton X‐100, incubated with anti‐γH2AX (Cell Signaling Technology) antibody and detected by flow cytometry (Becton Dickinson).

### 2.5 Western blot

Cells were collected and lysed with RIPA buffer containing fresh protease inhibitor mixture (50 μg/mL aprotinin, 0.5 mmol/L phenylmethanesulfonyl fluoride (PMSF), 1 mmol/L sodium orthovanadate, 10 mmol/L sodiumfluoride and 10 mmol/L glycerolphosphate). Protein concentrations were quantified by BCA assay. Equal amounts of proteins were separated by SDS‐PAGE and electro‐transferred onto nitrocellulose membranes. The membranes were blocked with 5% non‐fat milk in TBST buffer (20 mmol/L Tris‐buffered saline and 0.5% Tween 20) for 1 hour at room temperature prior to incubation with specific antibodies: Ser1981‐phosphorylated ATM, Ser428‐phosphorylated‐ATR, Ser296‐phosphorylated‐Chk1, Thr68‐phosphorylated‐Chk2, Ser1524‐phosphorylated‐BRCA1(p‐BRCA1), BRCA1, Ser139‐phosphorylated histone H2AX (γH2AX), (Cell Signaling Technology); glyceraldehyde‐3‐phosphate dehydrogenase (GAPDH), poly (ADP‐ribose) polymerase (PARP), c23, p27 (Kip1) and p21 (Cip1) (Santa Cruz Biotechnology); CTSB (Abcam, UK) overnight at 4°C. Following washing with TBST and incubating with peroxidase‐conjugated appropriate secondary antibodies, immunoblot proteins were visualized by enhanced chemiluminescence detection system (Millipore) and exposed to X‐ray films.

### 2.6 Subcellular fractionation

Nuclear and cytoplasmic extracts were prepared by using the Nuclear Extract Kit (Active Motif) following the manufacturer's instructions. Briefly, 5 × 10^6^ cells per sample were trypsinized and washed in PBS with phosphatase inhibitors. The cytoplasmic fraction was collected by centrifugation at 13 400 *g* for 10 minutes after treating the cells with 1% NP‐40 in Hypotonic Buffer supplemented with PMSF and protease inhibitors. Nuclear stability was determined at the microscope by Trypan blue staining. Pellet (nuclear extract) was washed in PBS containing 0.05% NP‐40. Nuclear proteins were extracted in Complete Lysis Buffer supplemented with 1 mmol/L dithiothreitol (DTT), PMSF and protease inhibitors. Samples were incubated in buffer for 10 minutes, sonicated for 5 seconds and centrifuged at 13 400 *g* for 10 minutes. After protein quantification, 80‐100 μg of protein were loaded per well by SDS‐PAGE.

### 2.7 Neutral comet assays

To assess DNA double‐strand breaks (DSBs), neutral comet assays were performed using CometSlide assay kits (Trevigen). Briefly, PCa cells were treated with RD‐N (6 μmol/L) and were incubated at 37°C for 0‐24 hours. Cells were embedded in agarose, lysed and subjected to neutral electrophoresis. Immediately before image analysis, cells were stained with SYBR Green and visualized under a fluorescence microscope (Olympus, Japan). Olive comet moment was calculated by multiplying the percentage of DNA in the tail by the displacement between the means of the head and tail distributions, as described.^15^ We used the program CometScore software to calculate Olive Comet Moment. A total of 30 comets were analysed per sample in each experiment.

### 2.8 CTSB activity

Cathepsin B activity was measured by using the fluorogenic substrate Z‐RR‐AMC from the EMD Chemicals following the manufacturer's instructions. Briefly, 10^6 ^cells were lysed in Lysis Buffer (100 mmol/L phosphate buffer, pH 6; 0.1% polyethylene glycol (PEG); 5 mmol/L DTT; 0.25% Triton X‐100), substrates were added at 20 μmol/L final concentration in 100 μL Lysis Buffer in the presence or absence of inhibitors for CTSB (E64d, CA074Me). A total of 100 μg of protein extract was used per sample. Cleaved Z‐RR‐AMC substrate was detected by fluorescence reader (Exc: 380 nm; Emi: 460 nm).

### 2.9 Immunofluorescence

Cells growing in coverslips were fixed for 10 minutes in ice‐cold methanol/acetone (1:1), followed by three washes in PBS. After blocking in 3% BSA in PBS with 0.1% Triton X‐100 for 20 minutes, cells were incubated with CTSB, γH2AX or p‐BRCA1 antibodies overnight at 4°C, washed three times and incubated 1 hour at 37°C with secondary antibodies. After washing three times in PBS, cells were counterstained with 4',6‐diamidino‐2‐phenylindole (DAPI) and coverslips mounted on slides. Fluorescence images were captured using a confocal microscopy (Carl Zeiss, Germany).

### 2.10 Protein modelling

We used the known crystal structure of BRCA1 and CTSB for protein docking. Crystal structure of BRCA1 ring domain (PDB ID: 1JM7)^16^ and BRCT domains (PDBID: 1JNX)^17^ were docked to the structure of CTSB (PDB ID: 3K9M)^18^ by ZDOCK.^19^ Two sets of 2000 structure complexes were generated and ranked according to the ZRANK scoring function.^20^

### 2.11 Microscopy

To visualize chromatin condensation, we used Hoechst33342 or DAPI to stain DNA in the nuclei. Briefly, PC3 cells cultured on cover glasses were incubated with 5 μg/mL Hoechst33342 or DAPI for 15 minutes. The cells were then washed with PBS and nuclear fluorescence was detected using fluorescence microscope (Olympus). Alternatively, apoptotic cells were identified using an in situ cell death detection TUNEL kit (Roche). The staining was performed according to manufacturer's instruction and observed using fluorescence microscope (Olympus).

### 2.12 Transfection

siRNA to human CTSB, BRCA1 and scrambled siRNA were purchased from Invitrogene. siRNA was transfected using siRNA double‐stranded oligonucleotides by Lipofectamine 2000. Knockdown of CTSB or BRCA1 was confirmed by immunostaining with CTSB or BRCA1 antibody. The cDNA sequence of CTSB was PCR amplified from the pEGFP CTSB plasmid, and then cloned into the pcDNA 3.1 vector and pEGFP N1. the truncated CTSB variants (ΔCTSB) were generated by Quick Change. The following primers were used: The pcDNA CTSB: the forward primer: 5ʹ‐CTAGCTAGCATGTGGCAGCTCTGGG‐3ʹ, the reverse primer: 5ʹ‐CCCCTTAAGATCGGTGCGTGGAATTCC‐3ʹ; the pcDNA CTSB‐NLS: the forward primer: 5ʹ‐CTAGCTAGCATGTGGCAGCTCTGGG‐3ʹ, the reverse primer: 5ʹ‐CCCCTTAAGATTGTATCCGTAGTGCTTG‐3ʹ; the pcDNA CTSB(Δ278), the forward primer: 5ʹ‐TGATGGGTGGCGAGGCCATCCGCAT‐3ʹ, the reverse primer: 5ʹ‐ATGCGGATGGCCTCGCCACCCATCA‐3ʹ; the pcDNA CTSB(Δ298), the forward primer: 5ʹ‐GCTGGTTGCCGCCTCCTGGAACAC‐3ʹ, the reverse primer: 5ʹ‐GTGTTCCAGGAGGCGGCAACCAGC‐3ʹ. The pEGFP CTSB‐NLS: the forward primer: 5ʹ‐CCGCTCGAGATGTGGCAGCTCTGGGC‐3ʹ, the reverse primer 5ʹ‐CGCGGATCCGGATTGTATCCGTAGTGCT‐3’; transfections of PC3 cells were performed in 6‐well plates. The cells were transfected with equal amounts of pcDNA 3.1/CTSB/ΔCTSB/CTSB‐NLS; pEGFP CTSB/CTSB‐NLS; pcDNA 3.1/BRCA1 constructs using Lipofectamine 2000.

### 2.13 Immunohistochemical analysis

CTSB, p‐BRCA1 and γH2AX protein expression were evaluated by immunohistochemistry on tumour sections using CTSB, p‐BRCA1 and γH2AX‐specific antibody. The sections were deparaffinized first. For antigen retrieval, the sections were boiled in 10 mmol/L citrate buffer (pH 6.0) for 30 minutes, and endogenous peroxidase activity was blocked using 3% H2O2 for 5 minutes. The sections were then blocked in antibody diluent for 1 hour at room temperature. CTSB, p‐BRCA1 and γH2AX antibodies were diluted 1:200 in blocking solution, and sections were incubated overnight at 4°C. After washing, the slides were incubated with anti‐goat biotinylated antibody for 30 minutes at room temperature. Sections were counterstained with haematoxylin, dehydrated and permanently mounted.

### 2.14 Statistical analysis

The values represent the mean ± SD for triplicate experiments. Statistical differences were assessed using an unpaired Student's *t* test and one‐way anova *P* < 0.05 was considered statistically significant.

## 3 RESULTS

### 3.1 RD‐N induces DNA damage in PCa cells

**Figure 1:**
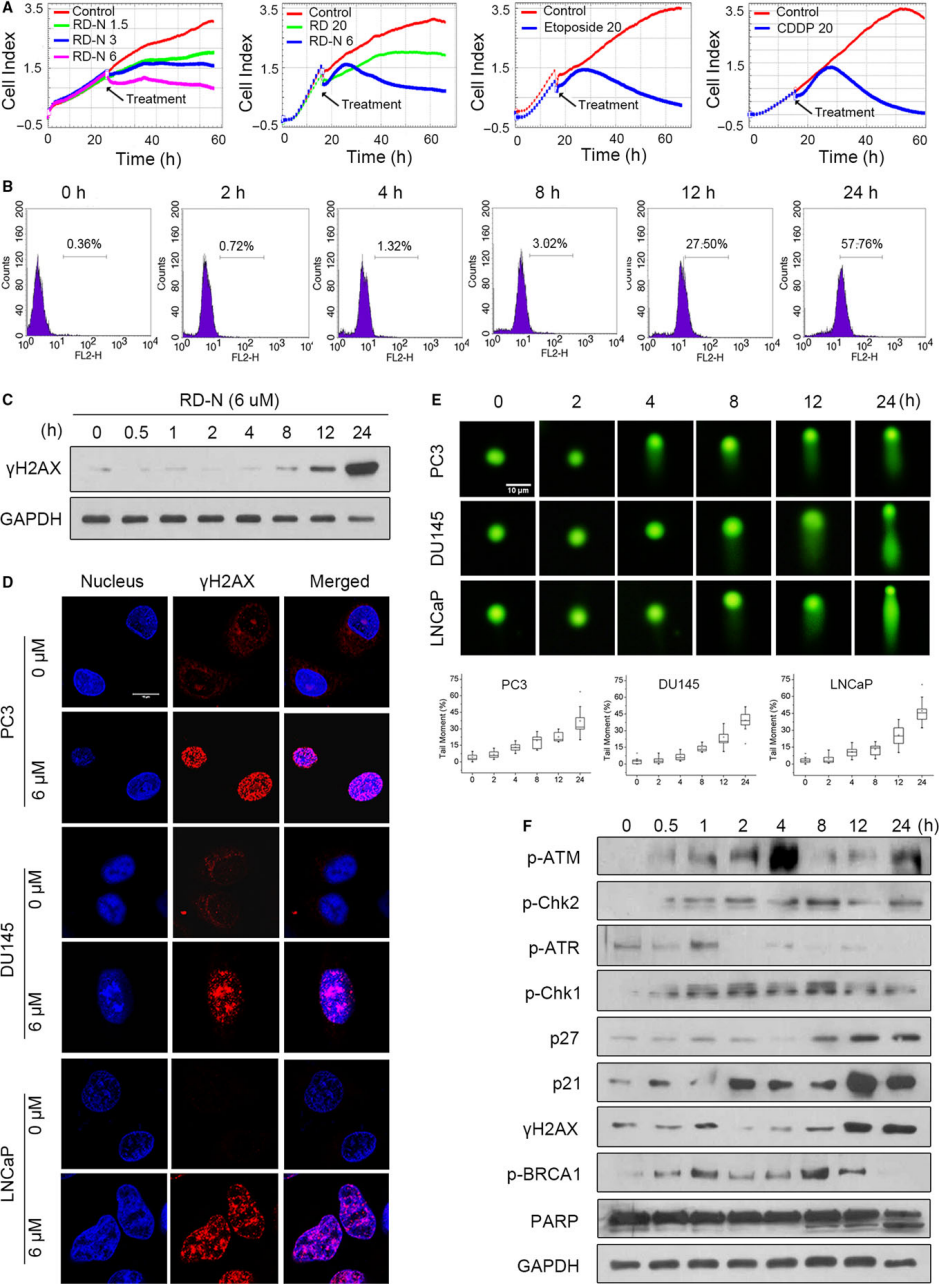
RD‐N induces cell death by DNA damage in PC3 cells. (A) PC3 cells were plated 10 000 cells/well into E‐plate 16. Cells were then treated with RD‐N, RD, etoposide or cisplatin as indicated and analysed using a xCELLigence RTCADP instrument. (B) Kinetics of the H2AX phosphorylation induced by RD‐N in PC3 cells. Cells were treated with RD‐N (6 μmol/L) for 24 h, and H2AX Ser139 phosphorylation was assessed by flow cytometry. (C) γH2AX immunoblotting detection in total extracts obtained at different times from prostate cancer (PCa) cells. (D) Immunofluorescent staining of γH2AX detected in PCa cells treated with RD‐N (6 μmol/L) for 24 h and stained with DAPI (to visualize nuclei) or a specific γH2AX antibody. Representative cells are shown. Scale bar, 10 µm. (E) Analysis of DNA strand breaks by a neutral comet assay performed in PCa cells after treatment with RD‐N (6 μmol/L) for indicated times. Representative comet images and percentage of cells with a tail (comet +) are shown (n = 30). Scale bar, 10 µm. (F) Changes of DNA damage proteins in RD‐N‐treated cells were analysed by immunoblotting. Results are representative of three independent experiments

We first validated the pro‐apoptotic effect of RD‐N on PC3 cells using the xCELLigence system. As shown in Figure 1A, RD‐N (6 μmol/L) sustainably reduced cell numbers followed by a quick growth period during treatment, displaying very similar growth response to those of etoposide (20 μmol/L) and cisplatin (Pt, 20 μmol/L). We thus selected etoposide as a positive control in further experiments. RD‐N triggered cell death was through induction of apoptosis as evidenced by an increase in the fraction of Annexin V‐FITC^+^/PI^+ ^cells and morphological changes in the nuclei (Figure S1A‐C).

We next determined whether DNA damage occurred during RD‐N–induced apoptosis in cancer cells. Flow cytometry analysis revealed a time‐dependent phosphorylation of H2AX at Ser139 (γH2AX), an indicator of DNA damage, upon treatment with RD‐N (Figure 1B). Similarly, the results in Figure 1C demonstrated that elevated γH2AX was noted at 8 hours, and became robust with longer treatment, time kinetic changes in the γH2AX were well matched with the induction of apoptosis in response to RD‐N (Figure S1A). Furthermore, immunofluorescence staining clearly showed that RD‐N promoted the formation of γH2AX nuclear foci, which usually characterizes DNA DSBs generation,^21^ in PC3 cells and another two PCa cell lines DU145 and LNCaP (Figure 1D). Comet assay supported the notion that DNA tail moments were hardly detectable within 2 hours in response to RD‐N, they became prominent at 12 hours and thereafter (Figure 1E). We therefore examined whether ATM‐Chk2 and ATR‐Chk1 signalling cascades, which are critical DNA damage signalling pathways activated by DSBs and single‐stranded DNA breaks,^22^ was attributable to RD‐N‐induced DNA damage. Kinetic studies showed that RD‐N activated ATM as early as 1 hour after treatment and persisted up to 4 hours as evidenced by accumulation of phospho‐ATM (Figure 1F). Activation of Chk2 (phospho‐Chk2 at Thr^68^) was sustained up to 8 hours and became weak after longer treatment (Figure 1F). Although the phosphorylation of ATR‐induced by RD‐N was also observed at 1 hour, it declined rapidly afterwards. Downstream Chk1 of ATR was activated until 12 hours in cells exposure to RD‐N. Accumulation of p27 (Kip1) and p21 (Cip1), which are regulated by Chk1/Chk2, was also observed in RD‐N‐treated cells. These results indicated that ATM/ATR cascades were initially activated to deal with the stress upon RD‐N treatment. Consequentially, γH2AX that is phosphorylated by ATM/ATR kinases was elevated at 12 hours and remained in a high level for up to 24 hours following RD‐N treatment, which was not due to the increase in H2AX total protein level. Activation of BRCA1 (phosphorylation at Ser^1524^), a critical molecule in the initial recruitment of repairing proteins and enzymes at the damaged breaks, was observed at 30 minutes and maintained up to 12 hours during RD‐N treatments but declined thereafter. Thus, an increase in the cleavage of PARP, a hallmark of apoptosis, was detectable at 8 hours and enhanced up to 24 hours, indicating a link between DNA damage and apoptosis in response to RD‐N. Thus, RD‐N induces apoptosis that is associated with induction of DNA damage in PCa cells.

### 3.2 RD‐N induces lysosomal CTSB translocation to the nucleus

**Figure 2:**
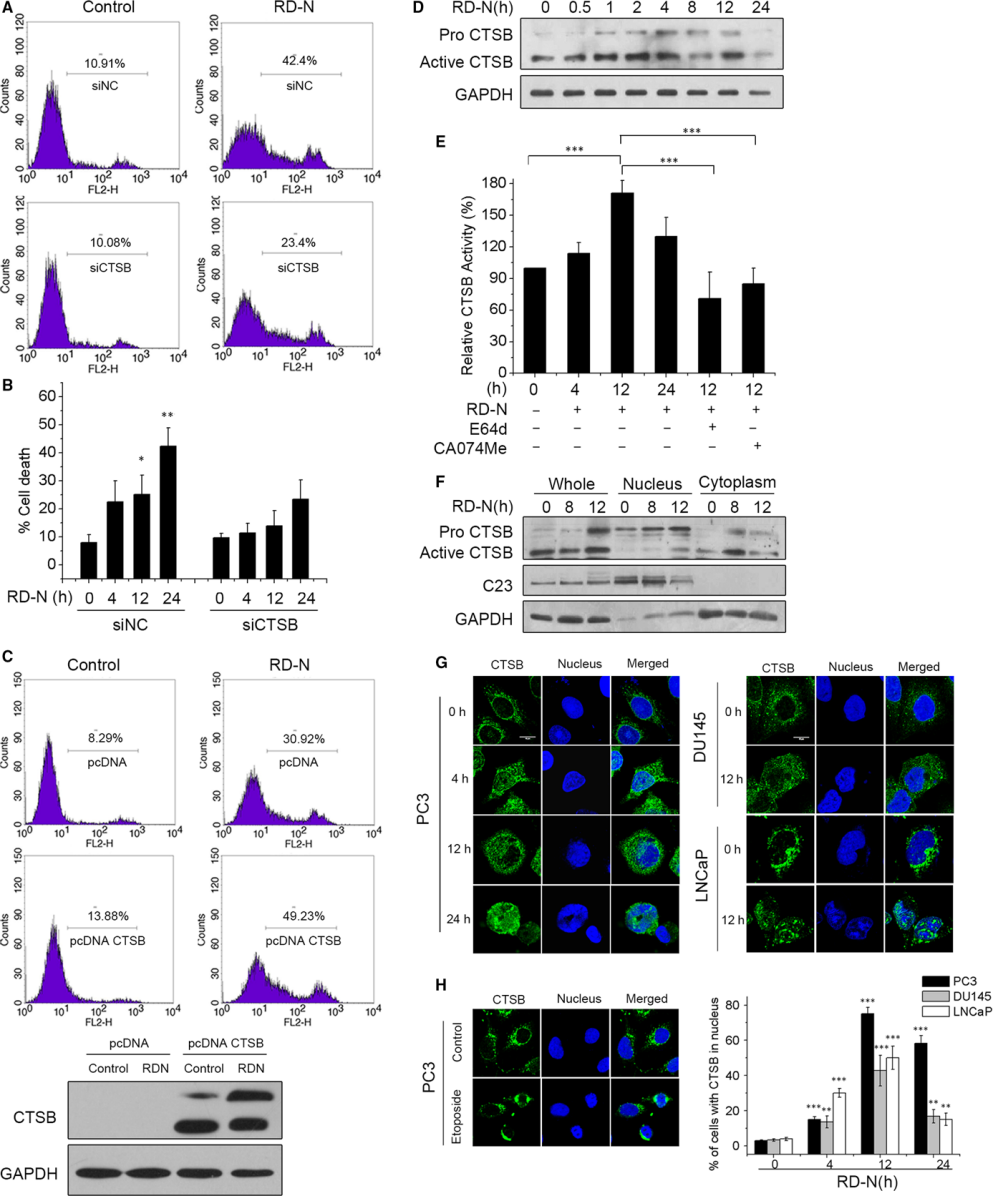
Cathepsin B (CTSB) is essential for RD‐N‐induced PCD by relocalization from the lysosomes to the nucleus in prostate cancer (PCa) cells. (A) Scramble siRNA and CTSB siRNA cells were untreated (control) or treated with RD‐N (6 μmol/L, 24 h), labelled with PI and analysed by flow cytometry. Representative cytofluorometric plots are shown. Percentages refer to PI‐positive staining. (B) Kinetic analysis of cell viability loss induced by RD‐N (6 μmol/L, 24 h) in scramble siRNA and CTSB siRNA PC3 cells. After the indicated times, cells were stained as in (A) and the frequency of PI‐positive labelling was recorded and expressed as a percentage. Data are the means of three independent experiments ±SD. *P < 0.05, **P < 0.01. (C) PC3 cells were transfected with the indicated expression plasmids and selected as described in Section 2. Then, cells were untreated (Control) or treated with RD‐N (6 μmol/L, 24 h). Cell death was analysed by PI. The expression level of CTSB was assessed by immunoblotting. Equal loading was confirmed by GAPDH assessment. Data are the means of three independent experiments ±SD. *P < 0.05, **P < 0.01. (D) CTSB immunoblotting detection in total extracts obtained at different times from PCa cells untreated or treated with RD‐N. (E) CTSB activities of cell extracts were measured. As a control, E64d (20 μmol/L) or CA074Me (10 μmol/L) were added in the culture medium 2 h before the harvest of cells. Each datum is an average of triplicate samples. *P < 0.05, ***P < 0.001. (F) Western blots showing the distribution of CTSB in PC3 cells. CTSB was detected in nuclear extracts obtained from PC3 cells treated with RD‐N. GAPDH and C23 were used to control. (G)Prostate cancer cells were treated with RD‐N (6 μmol/L), immunostained for CTSB detection (green) and examined by confocal microscopy. DAPI (blue) was used to visualize nuclei. Percentages refer to CTSB in nucleus cells. Scale bar, 10 µm. (H) PC3 were treated with etoposide and examined the relocalization of CTSB by confocal microscopy. Data are mean ± SD, n = 30, *P < 0.05, **P < 0.01 and ***P < 0.001

Our previous studies have shown that inhibition of CTSB by inhibitor CA074Me blocked RD‐N‐induced cell death,^1^ but the underlying molecular mechanisms remain unknown. The importance of CTSB in RD‐N‐induced apoptosis was assessed by knockdown of endogenous CTSB in PC3 cells in the presence of RD‐N. As shown in Figure 2A and B, CTSB depletion was markedly rescued RD‐N‐mediated apoptosis in PC3 cells. The fraction of apoptotic cells was 23.4% at 24 hours compared to 42.4% in scramble siRNA‐treated cells (Figure 2A,B). Also, CTSB silencing did not present similar amounts of chromatin condensation or TUNEL positivity to the levels of scramble siRNA‐treated cells in response to RD‐N (Figure S2A,B). In addition, we re‐introduced the CTSB into PC3 cells to confirm the role of CTSB in RD‐N‐mediated apoptosis. The results in Figure 2C showed that ectopic expression of CTSB exacerbated cell death induced by RD‐N. These findings demonstrated that CTSB is required for RD‐N‐mediated chromatinolysis and cell death. We therefore examined the response of CTSB in cells treated with RD‐N. As shown in Figure 2D, RD‐N treatment increased CTSB and its active form within 12 hours in PC3 cells, the level of CTSB declined at 24 hours treatment, the time point at which cellular apoptosis was predominantly induced (Figure S1A). As a proteolysis enzyme, the activity of CTSB in RD‐N‐treated PC3 cells was time‐dependently increased and peaked at 12 hours as indicated by the changes in the fluorescence of Z‐RR‐AMC, a substrate of CTSB (Figure 2E), which was correlated with the changes of CTSB protein levels. Of note, RD‐N‐stimulated CTSB activity was significantly reversed in the presence of cysteine protease pan inhibitor E64d and CTSB inhibitor CA074Me (Figure 2E), reinforcing the regulatory effect of RD‐N on CTSB activity. Moreover, RD‐N facilitated CTSB migration into the nucleus, the active form of CTSB were elevated in the nuclear fractions of PC3 cells treated by RD‐N for 12 hours (Figure 2F), while cytosolic CTSB was decreased under same conditions (Figure 2F). Immunofluorescence staining supported the observations that, in contrast to the untreated cells where CTSB was excluded from the nucleus and exhibited the typical lysosome pattern (Figure 2G), RD‐N treatment significantly facilitated translocation of CTSB in the nucleus. Similar results were also observed in those of DU145 and LNCaP cells upon RD‐N treatment (Figure 2G). However, etoposide had limited effect on the translocation of lysosomal CTSB to the nucleus (Figure 2H), supporting the special effect of RD‐N on CTSB. Thus, RD‐N was able to promote migration of CTSB to the nucleus, which may contribute to the induction of DNA damage and cell death.

### 3.3 CTSB is required for RD‐N induced DNA damage

**Figure 3:**
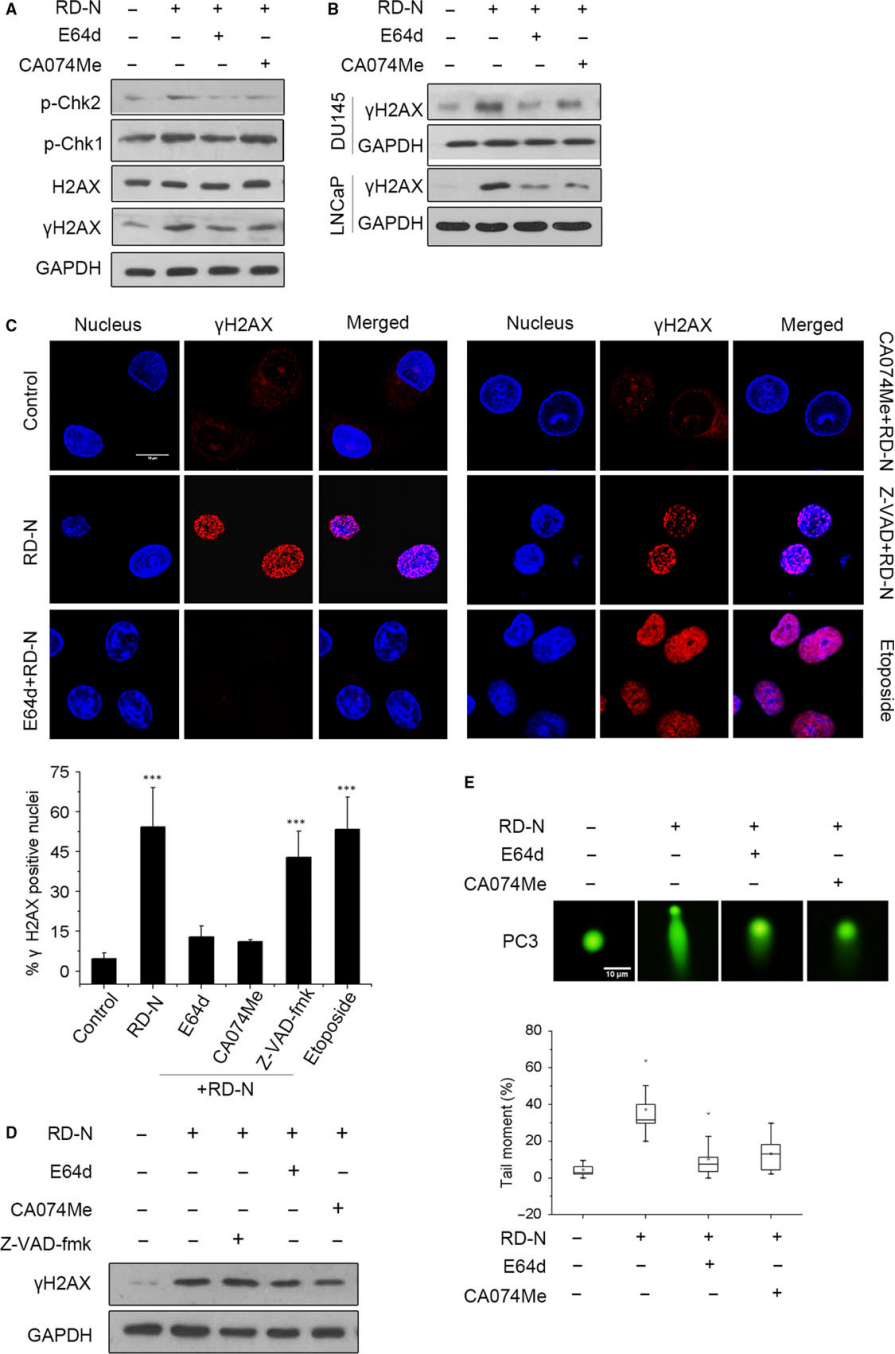
The DNA damage signalling pathways are blocked by cathepsin B (CTSB) inhibitors. (A) Exposed to RD‐N (6 μmol/L) untreated or treated with E64d (20 μmol/L) and CA074Me (10 μmol/L) for 24 h in PC3 cells, protein levels of DNA damage proteins were detected by immunoblotting. (B) Immunoblot analysis of expression levels of γH2AX in DU145, and LNCaP cells in response to RD‐N (6 μmol/L) pre‐incubated with E64d (20 μmol/L) and CA074Me (10 μmol/L), respectively. (C) Immunofluorescence staining of γH2AX foci in PC3 cells. Percentages refer to γH2AX foci positive in nucleus cells. Scale bar, 10 µm. Data are mean ± SD, n = 30, ***P < 0.001. (D) Immunoblot analysis of expression levels of γH2AX inPC3 cells exposure to RD‐N and various inhibitors (E64d: 20 μmol/L, CA074Me: 10 μmol/L, z‐VAD‐fmk: 20 μmol/L), respectively. (E) Neutral comet assays of PC3 cells treated with RD‐N in the presence orabsence of CTSB inhibitors for 24 h. Comet length was analysed by box and whisker plot method (n = 30). Scale bar, 10 µm

Since RD‐N induces the permeabilization of lysosomes and releases CTSB to the nucleus, we are promoted to investigate whether CTSB translocation to nucleus has a causative effect on DNA damage. PC3 cells were pre‐incubated with CTSB inhibitors (E64d and CA074Me) prior to RD‐N treatment. As shown in Figure 3A, inhibition of CTSB significantly reduced the levels of phospho‐Chk2, γH2AX in PC3 cells in the presence of RD‐N but had minimal effect on the phospho‐Chk1 and total H2AX, suggesting that ATM/ChK2 may be more important in RD‐N‐mediated effect which is consistent with the results in Figure 1F. Similarly, inhibition of CTSB also attenuated the H2AX activation in DU145 and LNCaP cells exposed to RD‐N (Figure 3B). In the presence of specific protease inhibitors, we found that inhibition of the lysosomal protease CTSB (E64d or CA074Me), but not of caspases (z‐VAD‐fmk), strongly prevented formation of γH2AX foci in the nucleus (Figure 3C) and decreased H2AX phosphorylation (Figure 3D). DNA strand‐break formation also provided evidence that inhibition of CTSB greatly alleviated RD‐N‐induced DNA damage (Figure 3F). The data demonstrated a key role for CTSB translocation to the nucleus in mediating the DNA damage induced by RD‐N.

### 3.4 Suppression of BRCA1 is essential for conferring CTSB‐induced DNA damage in response to RD‐N

**Figure 4:**
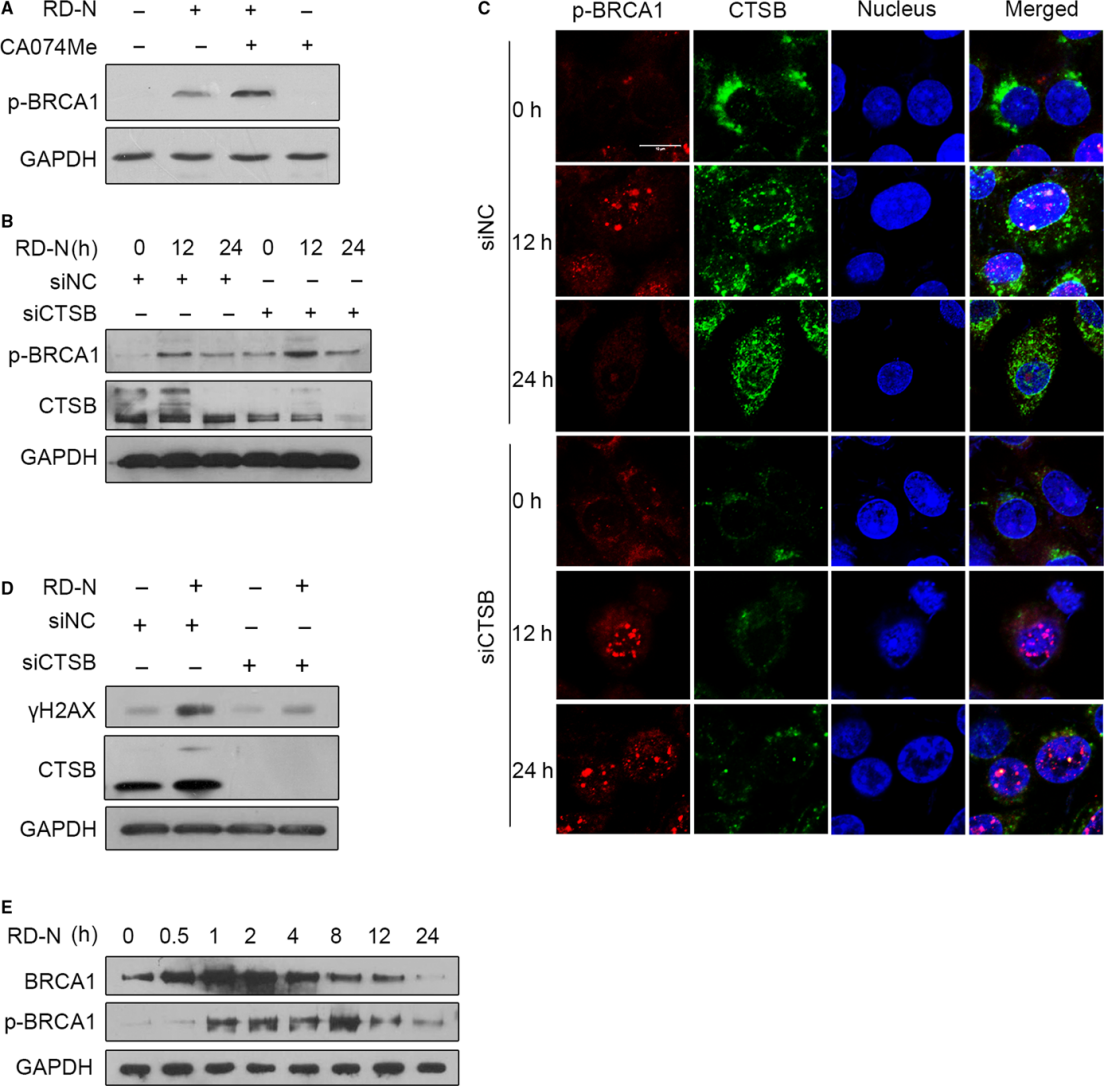
Cooperation between cathepsin B (CTSB) and breast cancer 1 protein (BRCA1) is required to promote DNA damage. (A) Prostate cancer (PCa) cells treated with RD‐N (6 μmol/L) and CA074Me were subjected to Western blot to detect the level of p‐BRCA1. (B) Detection of the levels of p‐BRCA1 after CTSB protein reduction using siRNA. siRNA knockdown of CTSB was performed in PC3 cells in combination with RD‐N (6 μmol/L) treatment for 12 and 24 h. (C) Immunofluorescence staining of p‐BRCA1 (red) and CTSB (green) in PC3 cells after CTSB protein reduction using siRNA. Nuclei were stained with DAPI (blue). siRNA knockdown of CTSB was performed in combination with RD‐N (6 μmol/L) treatment. Scale bar, 10 µm. (D) Effects of γH2AX after CTSB protein reduction using siRNA. siRNA knockdown of CTSB was performed in PC3 cells in combination with RD‐N (6 μmol/L) treatment for 24 h. γH2AX, CTSB were detected with the respective antibodies, as indicated. (E)The protein levels of BRCA1 after RD‐N treatment in PC3 cells

It was noted that p‐BRCA1 started to drop down at 12 hours and disappeared after 24 hours treatment, at which CTSB was evidenced in the nucleus associated with the increases in PARP cleavage in response to RD‐N (Figures 1F and 2). We hypothesized that CTSB acts as a proteolysis enzyme to induce DNA damage through down‐regulation of BRCA1 in cells exposed to RD‐N. The results in Figure 4A indicated that p‐BRCA1 was observed after 12 hours treatment with RD‐N, however, RD‐N‐induced BRCA1 phosphorylation was enhanced in the presence of CA074Me. Depletion of endogenous CTSB with specific targeting siRNA also significantly facilitated BRCA1 phosphorylation that was induced in PC3 cells after 12 hours treatment with RD‐N (Figure 4B). Immunofluorescence confocal microscopy further demonstrated that, in scramble siRNA cells, CTSB was confined to the lysosome, whereas p‐BRCA1 was localized in the nucleus (Figure 4C). Upon RD‐N treatment, CTSB moved to the nucleus and associated with the decreased p‐BRCA1 at 24 hours treatment (Figure 4C). However, CTSB deficiency caused an accumulation of p‐BRCA1 in the nucleus (Figure 4C), which attenuated H2AX phosphorylation exposed to RD‐N (Figure 4D). These findings supported that the migration of CTSB to the nucleus by RD‐N was important for down‐regulation of phosphor‐BRCA1 and induction of DNA damage. Since CTSB could process many proteins to be degraded,^23^, ^24^ we hypothesized that suppression of p‐BRCA1 ascribed to the degradation of BRCA1 by CTSB upon treatment with RD‐N. As shown in Figure 4E, total BRCA1 protein expression was steadily induced until 4 hours by RD‐N, and gradually faded thereafter. Accordingly, p‐BRCA1 was time dependently accumulated, and impaired after 8 hours treatments (Figure 4E), demonstrating the proteolysis ability of CTSB on BRCA1 degradation.

**Figure 5:**
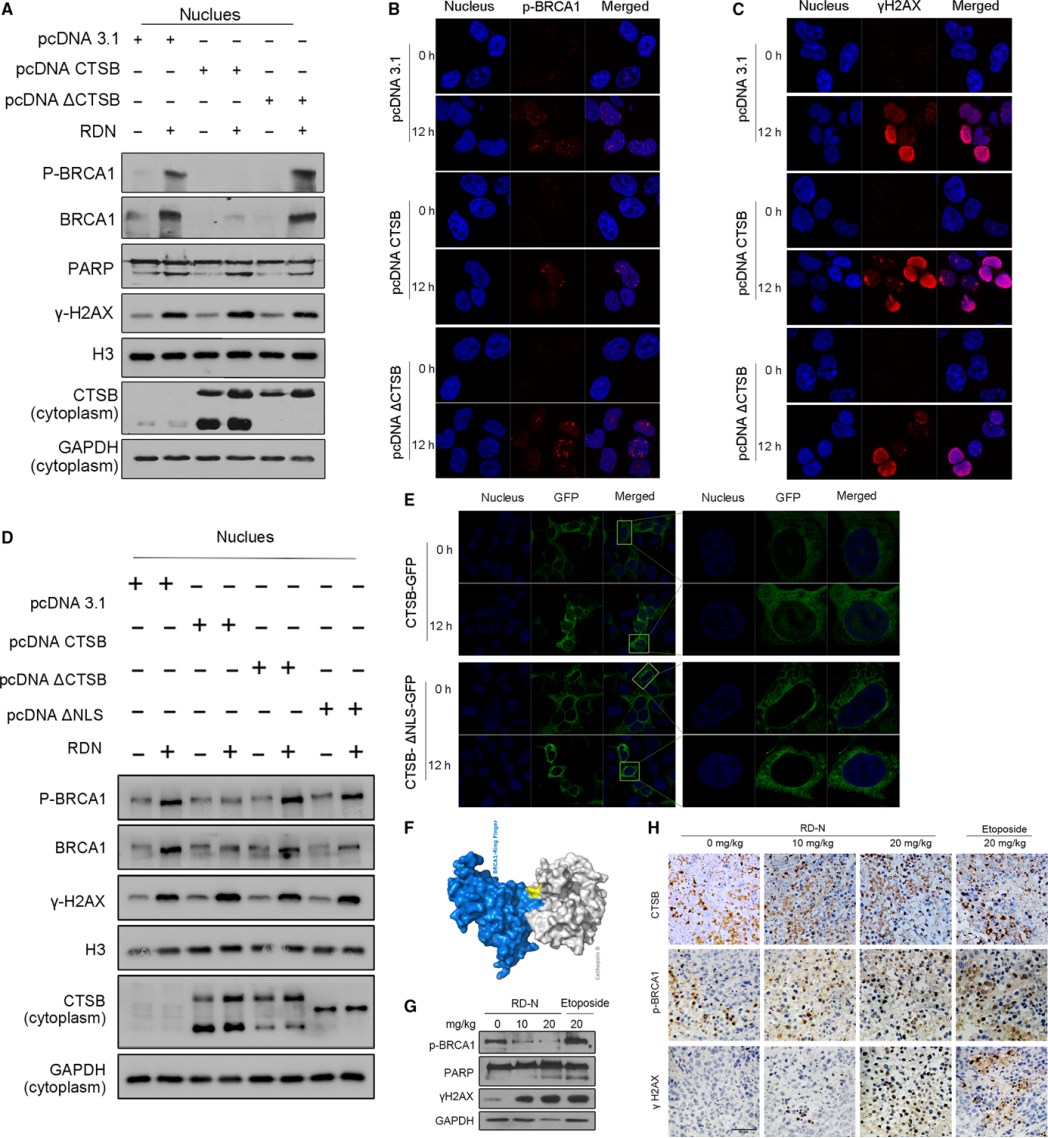
The function of the enzyme activity of cathepsin B (CTSB) is required to degrade breast cancer 1 protein (BRCA1). (A) Western blot to detect the level of BRCA1, p‐BRCA1, γH2AX, poly (ADP‐ribose) polymerase (PARP) in PCa cells treated with RD‐N (6 μmol/L) and plasmid (pcDNA 3.1, pcDNA CTSB, pcDNA ΔCTSB). (B) Immunofluorescence staining of γH2AX (red) in PC3 cells after CTSB protein overexpression with or without RD‐N (6 μmol/L) treatment. (C) Immunofluorescence staining of p‐BRCA1 (red), nuclei were stained with DAPI (blue). (D) Western blot to detect the level of BRCA1, p‐BRCA1, γH2AX in PCa cells treated with RD‐N (6 μmol/L) and plasmid (pcDNA 3.1, pcDNA CTSB, pcDNA ΔCTSB, pcDNA CTSB‐ΔNLS). (E) Prostate cancer cells were treated with RD‐N (6 μmol/L), the CTSB‐GFP and CTSB‐ΔNLS‐GFP was examined by confocal microscopy. DAPI (blue) was used to visualize nuclei. Percentages refer to CTSB in nucleus cells. Scale bar, 10 µm. (F) The model of docking between CTSB and BRCA1. (G) Western blot to detect the level of CTSB, p‐BRCA1 and γH2AX in RD‐N‐treated tissues of a PC3M‐luc‐C6 xenograft mouse model. Scale bar, 50 µm. (H) Immunohistochemical shows the expression of p‐BRCA1, γH2AX and PARP from tumour tissues in response to RD‐N and etoposide in vivo

The ability of CTSB on BRCA1 was further examined in cells transfected with a CTSB expression plasmid. As shown in Figure 5A, p‐BRCA1 was induced in the nucleus after 12 hours treatment which was consistent with the observation in Figure 1F, however, overexpression of CTSB significantly reduced the levels of BRCA1 and p‐BRCA1 in cells exposed to RD‐N (Figure 5A). Immunofluorescence analysis confirmed that forced expression of CTSB markedly abolished p‐BRCA1 in the nucleus (Figure 5B), and enhanced γH2AX foci in RD‐N‐treated cells (Figure 5C). To determine whether the effect of CTSB on BRCA1 relies on its proteolytic activity, we constructed a mutant CTSB expression plasmid lacking enzyme domain, resulting in an enzyme‐inactivated product^25^. Expression of enzyme‐negative CTSB (ΔCTSB) predominantly reduced its catalytic activity as demonstrated by losing the active bands, importantly, the level of phosphor‐BRCA1 was restored in cells when expression of ΔCTSB in the presence of RD‐N (Figure 5A). Also, forced expression of CTSB without enzyme domain was able to alleviate the PARP cleavage and γH2AX expression when compared to that of wild type of CTSB (Figure 5A). Immunofluorescence staining revealed that overexpression of CTSB markedly reduced phosphor‐BRCA1 that was induced in cells by RD‐N at 12 hours treatment (Figure 5B), which was associated with an increase in DNA damage as indicated by elevated γH2AX foci (Figure 5C). However, ΔCTSB lost its ability to reduce RD‐N‐activated BRCA1 which in turn attenuated DNA damage triggered by RD‐N, compared to the CTSB plus RD‐N under same conditions (Figure 5B,C). These results strengthened the importance of proteolytic activity of CTSB in the suppression of BRCA1. To further determine whether nuclear localization of CTSB plays a role in the degradation of BRCA1, we constructed a mutant CTSB expression plasmid lacking nuclear localization signal (CTSB‐ΔNLS).^26^ Expression of CTSB‐ΔNLS predominantly reduced its nuclear localization upon treatment with RD‐N (Figure 5E), importantly, the level of phosphor‐BRCA1 was restored in cells when expression of CTSB‐ΔNLS in the presence of RD‐N (Figure 5D). Also, forced expression of CTSB‐ΔNLS without nuclear localization signal was able to reduce γH2AX expression when compared to that of wild type of CTSB (Figure 5D). Finally, the available crystal structures of BRCA1 ring domain and BRCT domains (PDB ID: 1JNX) and CTSB (PDB ID: 3K9M) were used to predict the potential interaction sites. Docking images indicated that the BRCA1 ring domain was bound to the catalytic cysteine residue of CTSB, which strongly supported the observations that hydrolysis region of CTSB is required for reducing p‐BRCA1 protein level (Figure 5F). However, it is difficult to confirm the binding of the endogenous CTSB to p‐BRCA1 by immuneprecipitation, probably due to the fast degradation of a substrate when an enzyme bound. Therefore, inhibition of BRCA1 by CTSB was important for RD‐N‐mediated DNA damage. The changes in CTSB, p‐BRCA1 and γH2AX were further examined in animal tissue samples after treatment with RD‐N. As shown in Figure 5H, in contrast to the tumour samples that showed detectable p‐BRCA1 in etoposide‐treated animals, RD‐N noticeably inhibited the expression of p‐BRCA1, which was correlated with positively stained CTSB and elevated γH2AX in the samples. Western blotting also confirmed the observations that RD‐N suppressed p‐BRCA1 and induced H2AX phosphorylation, leading to the enhanced DNA damage in mice (Figure 5G). Thus, inhibition of BRCA1 by RD‐N was important in CTSB‐stimulated DNA damage.

### 3.5 BRCA1‐deficient cells are sensitive to RD‐N or combined with cisplatin

**Figure 6:**
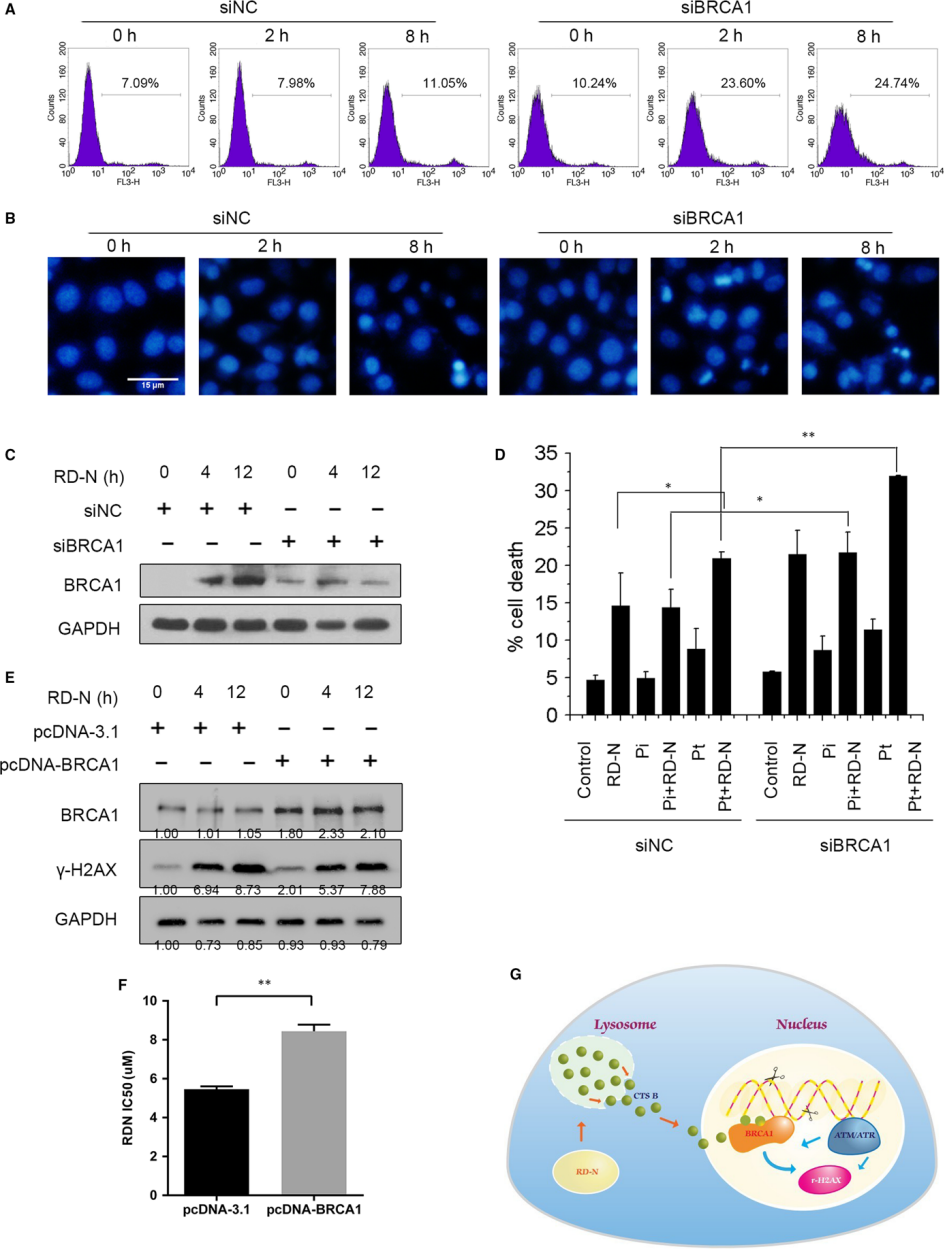
Breast cancer 1 protein (BRCA1) down‐regulation is essential for RD‐N‐induced cell death. (A) Scramble siRNA and BRCA1 siRNA cells were untreated (control) or treated with RD‐N (6 μmol/L, 8 h), labelled with PI and analysed by flow cytometry. (B) Scramble siRNA and BRCA1 siRNA PC3 cells were treated with RD‐N (6 μmol/L) and stained with DAPI to visualize nuclei. Scale bar, 15 µm. (C) PC3 cells were transduced with an siRNA for depletion of BRCA1 (siBRCA1) or a control siRNA (siScramble) and BRCA1 levels were assessed by Western blot. (D) siScramble and siBRCA1 cells were incubated with poly (ADP‐ribose) polymerase inhibitor (Pi) or cisplatin and RD‐N, and the extent of cell death was assessed by flow cytometry. Data are the means of three independent experiments ±SD, *P < 0.05, **P < 0.01. (E) Western blot to detect the level of BRCA1, γH2AX in prostate cancer cells treated with RD‐N (6 μmol/L) and plasmid (pcDNA 3.1, pcDNA BRCA1). (F) The IC50 of RD‐N in PC3 cell with the plasmid of pcDNA3.1 and pcDNA‐BRCA1. (G) Model for RD‐N‐mediated cell death. Cathepsin B released from the lysosomes moved to the nucleus and promoted DNA damage by degrading BRCA1

Since RD‐N induced BRCA1 degradation through CTSB, we sought to determine whether down‐regulation of BRCA1, which is critical in maintaining genomic integrity by promoting homologous recombination (HR), would affect RD‐N mediated apoptosis of cancer cells. As shown in Figure 6A, knockdown of the endogenous BRCA1 resulted in much higher frequency of apoptotic cells after 8 hours treatment with RD‐N than those cells treated with RD‐N alone (24.74% vs 11.05%, Figure 6A,B). The level of BRCA1 at 8 hours was efficiently reduced as monitored by Western blotting (Figure 6C). We also assessed the effects of combination of RD‐N with cisplatin (Pt) or with PARP inhibitor (Pi), which are genetoxic agents, in BRCA1‐deficient cells. Indeed, depletion of BRCA1 sensitized cells to Pt and Pi, consistent with the previous reports.^27^, ^28^ More important, BRCA1‐deficient cells showed a marked increase in cell death compared to scramble siRNA‐treated cells in response to RD‐N plus Pt, or combined with Pi (Figure 6D). To determine whether the BRCA1 is involved in the induction of DNA damage by RDN, we transfected a BRCA1 expression plasmid into cells to determine whether the BRCA1 is involved in the induction of DNA damage in response to RDN. As shown in Figure 6E‐F, overexpression of BRCA1 can reverse DNA damage and cell death by RDN. Taken all together, we provided evidence that RD‐N resulted in cysteine protease CTSB translocation from the lysosomes to the nucleus, which in turn leading to the degradation of BRCA1 protein and defects in HR (Figure 6G).

## 4 DISCUSSION

Rapidly dividing and invasive cancer cells are strongly dependent on effective lysosomal function.^3^, ^29^ Furthermore, lysosomes also play an important role in cancer drug resistance by sequestering cancer drugs in their acidic environment, leading to a reducing of the drugs' effects.^30^ Targeting lysosomes therefore have great therapeutic potential in cancer, because it not only triggers apoptotic and lysosomal cell death pathways but also reverses drug resistance.^29^, ^31^ We provide evidence here that targeting the lysosome by RD‐N causes a cathepsin‐dependent apoptosis via induction of DNA damage. Enhanced translocation of CTSB to the nucleus by RD‐N promoted the degradation of phosphor‐BRCA1, failing to repair damaged‐DNA. Importantly, RD‐N significantly increased cisplatin cytotoxicity, because lysosomal transporters are shown to mediate cellular resistance to cisplatin.^32^ Despite the substantial investigations performed on CTSB, little is known about the mechanisms by which this protein promotes caspase‐independent apoptosis.^33^ CTSB may play two opposing roles in malignancy: as an executioner of apoptosis in cytotoxic signalling cascades and a mediator of tumour invasion.^34^, ^35^ In this study, we provided a novel mechanism for the pro‐apoptotic action of CTSB. Lysosomotropic RD‐N‐mediated apoptosis, at least in part, required the nuclear translocation of CTSB and BRCA1 rather than caspase activation. Together with our previous results,^1^ we propose a novel sequence of molecular events for CTSB‐dependent cell death; namely, RD‐N‐induced the translocation of CTSB from the lysosomes to the nucleus, where CTSB triggered DNA damage, and mediates degradation of BRCA1 to cause DNA repair defects. The degradation effect of CTSB on BRCA1 may be similar to the action of trypsin, but CTSB is a highly specific enzyme.^36^ Some studies have shown that CTSB may exert its digest effect intracellularly or extracellularly, depending on the cell type and location of CTSB.^37^, ^38^ Our study demonstrated that migration of CTSB to the nucleus was critical in mediating the DNA damage induced by RD‐N. Unlike to RD‐N, DNA‐damaging agent etoposide‐mediated apoptosis seems not require the nuclear redistribution of CTSB. Therefore, CTSB‐mediated DNA damage was variable in response to agents.

It has been known that acidic proteases, but not the proteasome or calpain, degradeBRCA1 in DU145, SKBR3 and MCF7 cells,^39^ however, no specific cathepsin was identified for the degradation. We showed here that CTSB could negatively regulate BRCA1 expression, and enzyme‐negative domain of CTSB failed to suppress the phosphor‐BRCA1. Although CTSB and phospho‐BRCA1 did not co‐immunoprecipitate in RD‐N‐treated cells, probably it is difficult to get an enzyme‐substrate complex, the available crystal structures of BRCA1 ring domain was able to bind to the catalytic cysteine residue of CTSB. We may conclude that degradation of BRCA1 by CTSB, at least partially, leads to increased level of γH2AX in cells treated with RD‐N. In response to DNA damage, cells could undergo apoptosis if the damaged DNA fails to be repaired.^40^, ^41^ Thus, the specific redistribution of CTSB in the nucleus appears to be essential to the CTSB/BRCA1‐axis action, leading to the CTSB‐dependent cell death in response to RD‐N.

One of the first responses to the production of DSBs in DNA is the rapid generation of phosphorylated H2AX at Ser139 near the DNA break point. Our data showed that H2AX is phosphorylated after RD‐N treatment for 12 hours, at which the phosphorylation is crucial for CTSB‐dependent DNA damage. Each type of DNA damage elicits a specific cellular repair response.^42^, ^43^ BRCA1 is a nuclear tumour suppressor that is critical for resolving DSBs and interstrand crosslinks by HR. BRCA1‐deficient cancers are highly sensitive to chemotherapeutic agents.^44^, ^45^ In our study, BRCA1 was rapidly activated upon RD‐N treatment, followed by significant down‐regulation after 12 hours exposure, indicating the involvement of BRCA1 in RD‐N‐mediated DNA damage. As expected, BRCA1 depletion resulted in enhancement of cell death induced by RD‐N. Importantly, targeting lysosomes by RD‐N caused genomic instability, which in turn increased cell response to cisplatin in cells, and more evident in BRCA1‐deficient cells.

In summary, targeting lysosomes by RD‐N engages a novel cell death pathway that can be independent of classical apoptotic pathways. This offers a novel option for induction of cell death in tumour cells resistant to DNA damage agents. In particular, the use of RD‐N as a single agent or in combination with DNA damage agents is a potential strategy for cancer treatment, especially for HR‐deficient tumours. Lysosomes are therefore promising drug targets because lysosomal sequestration of chemotherapeutics is implicated in mediating drug resistance. This study might prove a promising agent that targets lysosomes with a novel mechanism for PCa treatment.

## CONFLICT OF INTEREST

None declare.

## AUTHOR CONTRIBUTIONS

Y.W., H.N. carried out experiments, made figures and wrote the manuscript; Z.H., M.Z., L.W., L.H. and L.Q. analysed the data; K.T. performed data analyses and reviewed the manuscript; H.Y., H.L. supervised and designed the research, analysed and interpreted the data, revised and polished the manuscript.
