# Supplementary figures and images for: Targeting the lysosome by an aminomethylated Riccardin D triggers DNA damage through cathepsin B‐mediated degradation of BRCA1
Source: J Cell Mol Med. 2018 Dec 18;23(3):1798–812. doi: 10.1111/jcmm.14077 (PMC6378192; doi:10.1111/jcmm.14077)

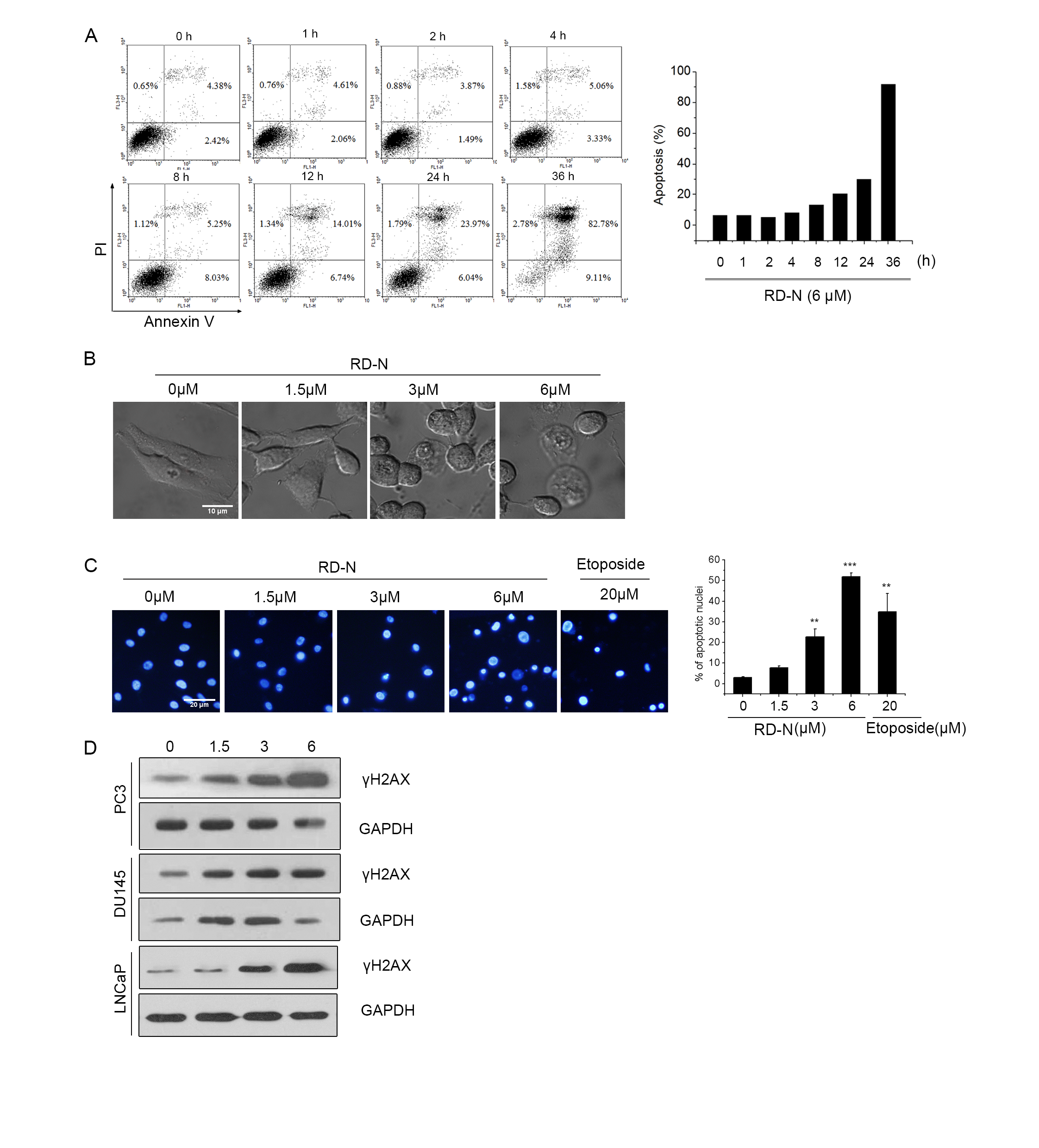

Supplement: Supplementary file 1 [file JCMM-23-1798-s001.tif]

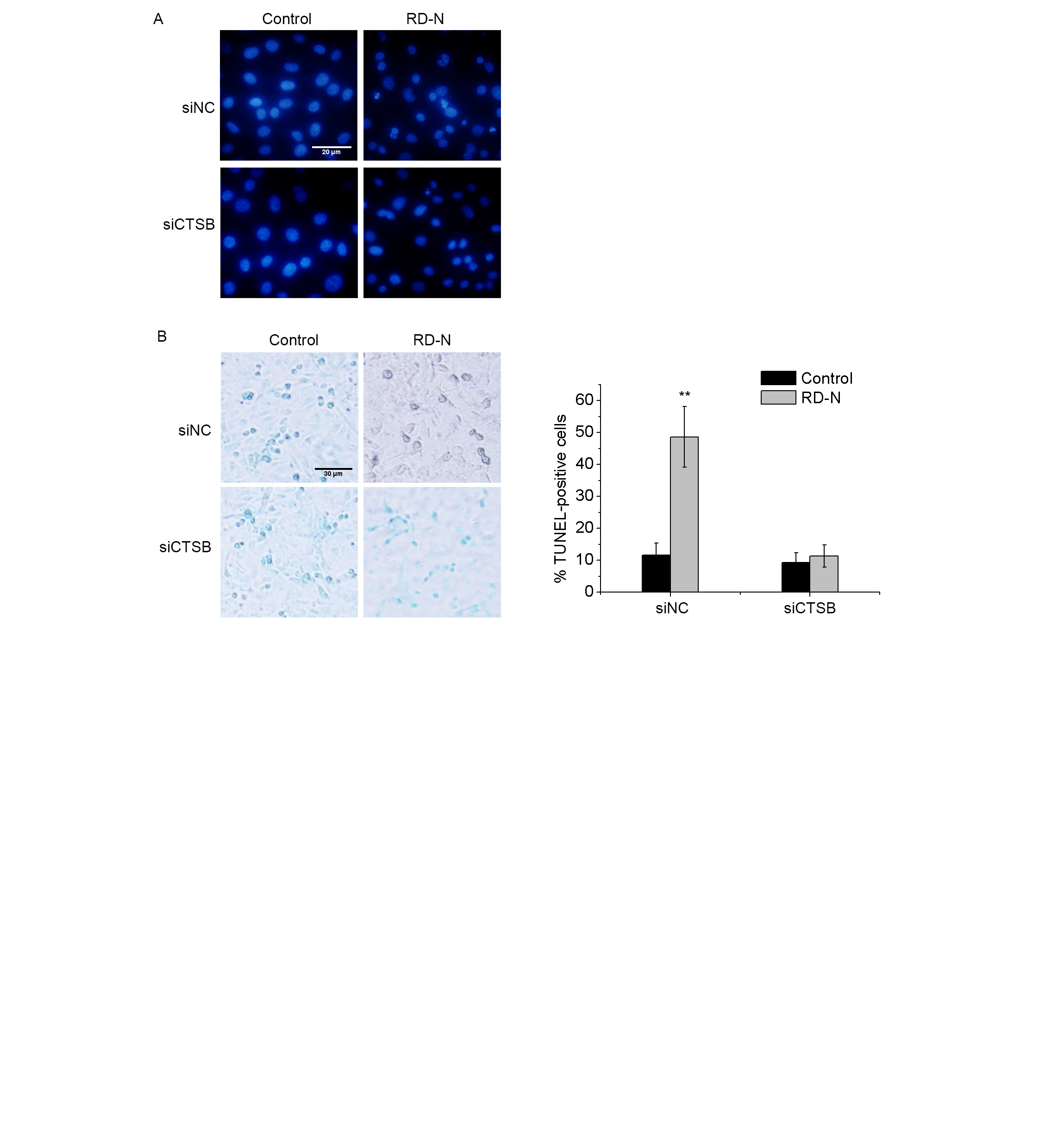

Supplement: Supplementary file 2 [file JCMM-23-1798-s002.tif]
