# Supplementary material for: Targeting the lysosome by an aminomethylated Riccardin D triggers DNA damage through cathepsin B‐mediated degradation of BRCA1
Source: J Cell Mol Med. 2018 Dec 18;23(3):1798–812. doi: 10.1111/jcmm.14077 (PMC6378192; doi:10.1111/jcmm.14077)
Supplement: Supplementary file 3 [file JCMM-23-1798-s003.doc]

**Supplemental figures legends**

Supplemental Figure 1

(A) PC3 cells were treated with 6 μM RD-N for 0–24 h. Cells were collected and stained with Annexin V-FITC and PI, and subjected to flow cytometry. (B) Cell growth inhibition activity of RD-N after treatment with various concentrations of 1.5–6 μM for 24 h on PC3 cells. Scale bar, 10 µm. (C) Quantification of condensed nuclei detected with Hoechst33342 staining after RD-N or etoposide treatment for 24 h. The percentage of cells with condensed nuclei was counted. Scale bar, 20 µm. (D) Western blot to detect the level of γH2AX in prostate cancer cells treated with RD-N (6 μM). Data are the means of 3 independent experiments ± SD, **P< 0.01 and ***P< 0.001.

Supplemental Figure 2

(A) Scramble siRNA and CTSB siRNA PC3 cells were treated with RD-N (6 μM) and stained with DAPI to visualize nuclei. Representative nuclei from untreated (control) or RD-N-treated cells are shown.Scale bar,20 µm. (B) Scramble siRNA and CTSB siRNA PC3 cells were treated with RD-N (6 μM) as in (A), representative microphotographs from each treatment are shown. Scale bar, 30 µm.
